# Sharing Marks: H3K4 Methylation and H2B Ubiquitination as Features of Meiotic Recombination and Transcription

**DOI:** 10.3390/ijms21124510

**Published:** 2020-06-25

**Authors:** Joan Serrano-Quílez, Sergi Roig-Soucase, Susana Rodríguez-Navarro

**Affiliations:** Gene Expression and RNA Metabolism Laboratory, Instituto de Biomedicina de Valencia (CSIC), Jaume Roig, 11, 46010 Valencia, Spain; jserrano@ibv.csic.es (J.S.-Q.); serroisoupv@gmail.com (S.R.-S.)

**Keywords:** meiosis, recombination, DSB, transcription, COMPASS, histone, PAF1c, methylation, ubiquitination

## Abstract

Meiosis is a specialized cell division that gives raise to four haploid gametes from a single diploid cell. During meiosis, homologous recombination is crucial to ensure genetic diversity and guarantee accurate chromosome segregation. Both the formation of programmed meiotic DNA double-strand breaks (DSBs) and their repair using homologous chromosomes are essential and highly regulated pathways. Similar to other processes that take place in the context of chromatin, histone posttranslational modifications (PTMs) constitute one of the major mechanisms to regulate meiotic recombination. In this review, we focus on specific PTMs occurring in histone tails as driving forces of different molecular events, including meiotic recombination and transcription. In particular, we concentrate on the influence of H3K4me3, H2BK123ub, and their corresponding molecular machineries that write, read, and erase these histone marks. The Spp1 subunit within the Complex of Proteins Associated with Set1 (COMPASS) is a critical regulator of H3K4me3-dependent meiotic DSB formation. On the other hand, the PAF1c (RNA polymerase II associated factor 1 complex) drives the ubiquitination of H2BK123 by Rad6-Bre1. We also discuss emerging evidence obtained by cryo-electron microscopy (EM) structure determination that has provided new insights into how the “cross-talk” between these two marks is accomplished.

## 1. Overview of Some Molecular Events Occurring during Meiosis in Yeast

The budding yeast *Saccharomyces cerevisiae* has a strong dependence on the presence of nutrients in its surroundings. As long as there is enough nutrient availability, cells proceed to mitosis, allowing them to proliferate by budding [1]. Provided that diploid cells are subjected to some nutrient starvation, they undergo a differentiation process called sporulation, where meiosis plays an essential role [2]. This specialized cell division process generates four haploid daughter cells by means of two consecutive cell divisions [3]. Meiosis is piloted by a cascade of transcriptional events as soon as mating-type and nutrition signals are brought together [4].

### 1.1. Transcriptional Events Leading to Meiosis

The ability to sporulate, which is unique to diploid cells, requires the expression of both MATa and MATα alleles. The products of this locus give rise to an a1/α2 heterodimer, without which sporulation is unable to take place [1]. Nonetheless, the decision to undergo this process depends mainly on several extracellular factors, including the presence of nitrogen and glucose, among others.

Most of these elements are able to control the transcription of the master regulator Ime1. The regulation of the expression of this factor is mediated by the Rme1 repressor [5], which is inhibited in the presence of the a1/α2 heterodimer that is expressed in haploid cells [6]. Consequently, in these cells, the a1/α2 heterodimer is able to bind to an Rme1 Repressor Element (RRE) situated within the promoter region of *IME1*. This promoter is much larger than that of the majority of yeast genes and contains regulatory elements for all the different factors that could potentially affect *IME1* expression. These regulatory elements include binding sites for transcription factors responding to mating type, nitrogen deprivation, and others [7]. In haploid cells, Rme1 is able to activate the expression of a long non-coding RNA (lncRNA), called *IRT1*, which nearly encompasses the totality of the *IME1* promoter. This lncRNA acts as a *cis*-element, inhibiting the binding of transcription factors to this region [8].

Furthermore, not only does mating affect the Rme1-dependent pathway, but it modulates the expression of the RNA-methyltransferase Ime4, needed for correctly inducing *IME1* [9]. What makes this factor especially interesting is the fact that its transcripts are different in concordance to the cell type; haploid cells express a non-coding anti-sense *IME4* RNA (known as *IME4-AS* or *RME2*), whereas in diploids, its anti-sense transcription is impeded by the aforementioned a1/α2 heterodimer. As a result, the Ime4 protein is produced, activating *IME1* by the N6-adenosine methylation of its RNA [10,11,12].

Once Ime1 is activated, a first transcriptional wave is triggered, consisting of the so-called “early” genes, which exhibit in their promoters a common regulatory element: the *URS1* site [13,14]. This *cis*-element is the target of the Ume6 protein, which after binding to the *URS1* site displays the capacity of repressing the transcription of these genes during growth, by the recruitment of the Rpd3/HDAC complex and the chromatin remodeler Isw2 [13,15,16,17].

The Ume6 protein has the ability to act as a repressor, but also as an activator. The conversion of Ume6 from repressor to activator was initially thought to be due to impeded interaction with the Rpd3/HDAC complex by the presence of Ime1 [18]. However, more recent studies have shown that this conversion is mediated in a different way. In order to bring about the expression of early genes, Ume6 must undergo a two-step degradation process, mediated by the anaphase-promoting complex/cyclosome (APC/C) ubiquitin ligase [19,20]. The function of Ume6 is regulated by its acetylation by the acetyltransferase Gcn5, belonging to the Spt-Ada-Gcn5 acetyltransferase (SAGA) complex [21], as well as by deacetylation by Rpd3 [22]. Ume6 acetylation reduces its DNA-binding activity, which provokes its disassociation from promoters and triggers its targeted destruction. As a consequence, transcription of early genes is activated [23]. Later on, when Ime1 is present, it binds to Ume6, which is further acetylated by Gcn5 in a second lysine cluster, which promotes its second destruction step, enabling the full activation of early genes [22,23]. As previously mentioned, these genes present a common *URS1* site to which Ume6 is bound, and whose expression is indispensable for the entrance into the premeiotic S phase [24]. Among these early genes, we can find *IME2*, a key regulator in meiosis by mediating Ime1 stability; *INO1*, which is involved in lipid biosynthesis; or *SPO13*, which is linked to sister chromatid cohesion [13,16,23,25].

### 1.2. Meiotic Recombination: DSB Formation, Repair, and Distribution

The segregation of homologous chromosomes occurs during the first meiotic division, while the second division is comprised of the separation of sister chromatids. The physical location where homologous chromosomes have exchanged their information is termed the chiasma. It is in this place where the spindle microtubules attach to kinetochores, generating tension. This tension is required for proper segregation at meiosis I [26]. It is in the first step where the process of interhomologous recombination becomes crucial, in order to guarantee genetically diverse gametes [26,27]. Problems during this recombination may give rise to germline mutations or even aberrant chromosome arrangements in gametes [28].

**Figure 1:**
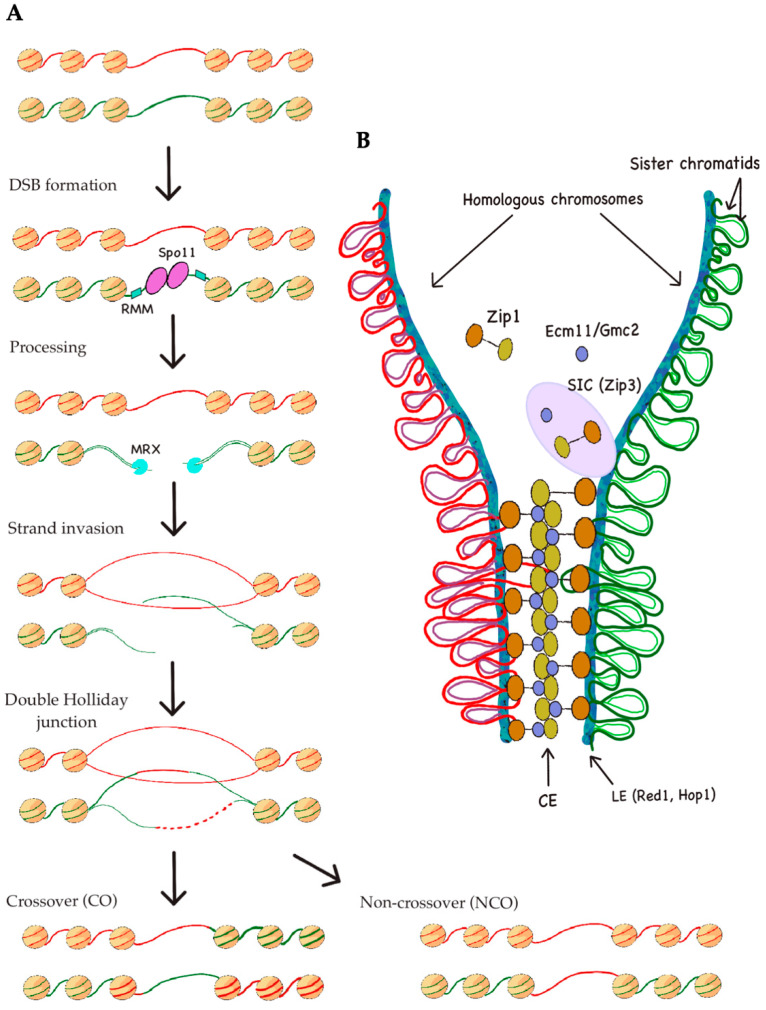
Overview of the recombination process during meiosis. (**A**) The mechanism of meiotic recombination. Double-strand breaks (DSBs) are formed by Spo11 and stabilized by the Mer2–Mei4–Rec114 (RMM) subcomplex; then they are processed by several factors, including the Mre11–Rad50–Xrs2 (MRX) complex, to yield single-stranded DNA. One of the strands invades the homologous chromosome, giving rise to a double-Holliday junction intermediate. This structure can be resolved, resulting either in crossover or non-crossover; (**B**) Synaptonemal complex (SC) assembly in yeast by Zip1. Zip1 protein rapidly polymerizes, which together with factors Ecm11 and Gmc2 localizes in the interior part of the complex (central element, or CE). Associations with the synapsis initiation complex (SIC), including Zip3, are necessary for a correct recruitment of the aforementioned factors. Proteins Red1 and Hop1 are responsible for lateral element (LE) formation, to which sister chromatids remain attached.

The recombination characterising meiosis consists of four consecutive steps (Figure 1A); (i) the initiation, where programmed DNA double-strand breaks (DSBs) are formed—a maximum of 200 per yeast nucleus [29,30]; (ii) the processing of these DSBs, giving rise to single-strand regions; (iii) a homologous repair of the DSBs, mainly by means of the homologous recombination pathway, as well as meiosis-specific modulators; and (iv) these double-Holliday junctions needing to be resolved and dissolved, thus mainly forming reciprocal crossovers (COs). In the context of meiosis, however, the majority of non-crossover events (NCO) arise from other pathways [31].

As stated, programmed DSBs are formed during the first meiotic division, a reaction catalyzed by the meiosis-specific, topoisomerase-like DNA transesterase Spo11 [32,33] (Figure 1A). Apart from this protein, nine DSB proteins have been described so far in *Saccharomyces cerevisiae* [34,35,36]. The majority of proteins associated to Spo11 form sub-complexes, including the Mer2–Mei4–Rec114 (RMM) subcomplex, which attaches to DSB sites in chromatin loops, permitting them to be cleaved by Spo11 [34]. Among these regulating factors, we can also encounter the Mre11–Rad50–Xrs2 (MRX) complex, Rec102, Rec104, and Ski8 [37].

After these steps, crossover recombination and homologous pairing are linked by the synaptonemal complex (SC), which implicates the multimeric assembly of coiled-coil proteins between aligned homologous chromosomes [38]. In spite of the mechanisms not being completely understood, the SC plays an important role for crossover recombination, and several links between the SC and recombination have been discussed [39]. Among its described subunits in yeast, we can find, on the one hand, Rec8, Red1, and Hop1, which conform the two parallel lateral elements (LEs) [40,41]. On the other hand, the central element (CE) proteins Ecm11 and Gmc2, and the traverse filament component Zip1, localize to centromeres in early prophase [42], mediating the coupling of homologous centromeres, and afterwards, the correct pairing of whole homologous chromosomes [43]. In order to properly build the SC, the assembly of the synapsis initiation complex (SIC) is urged, comprising the Zip2, Zip3, Zip4, and Spo16 proteins [44,45] (Figure 1B).

Once the recombination process has ended, the expression of Ndt80 implies entering into the middle phase [2]. The next main cytological event consists in dividing into four haploid cells. For this purpose, the spindle pole body (SPB), the sole microtubule-organizing centre in both budding and fission yeast, plays a key role. Its architecture is similar to the centrosome present in animal cells, and coordinates microtubule attachment and chromosome segregation [46]. SPBs duplicate twice: at the beginning of both meiosis I and II. The attachment of the spindle to the kinetochore is facilitated by the Dam1 (also named DASH) complex and carried out during metaphase, while chromosomes are pulled apart in anaphase [47,48]. In addition, the phosphorylation of some components of the SPB has been anticipated to stimulate the interaction with DSB to promote an efficient DNA repair [49].

In conclusion, DSB formation and repair are precisely regulated to ensure that this step is performed in a coordinated manner during meiotic recombination.

Nonetheless, the distribution of DSBs in *S. cerevisiae* does not occur at random; it is rather localized into specific regions, called recombination hotspots, where DSBs preferentially form. These short regions are, at the same time, contained within larger regions known as DSB-hot domains—these are opposed to DSB-cold domains, which mainly devoid of DSBs [30]. Among the mechanisms to control the distribution along the genome of these DSBs, chromatin accessibility becomes essential, allowing protein–DNA contacts that serve as docking sites for the recombination machinery [50]. To correctly accomplish it, several DNA-binding proteins, such as Atf-Pcr1, Bas1, or even Prdm9, in mammals, can participate in their regulation [51]. Of interest for this review, we would like to remark that these factors are able to incite some posttranslational modifications (PTMs) of histones, which in turn tempers chromatin disposition (see later section) [52].

## 2. Different Histone Modifications That Are Linked to Meiotic Events

As explained above, DSBs occur in DNA. In eukaryote cells, DNA is found wrapped around specialized proteins called histones, making up nucleosomes, the fundamental unit of chromatin [53]. The core particle of nucleosomes contains two copies of the four histones: H2A, H2B, H3, and H4, whose tails are susceptible to be modified by PTMs to regulate the chromatin state [52]. The involvement of these PTMs in different molecular events during meiosis appears to be undisputable, for it is one of the most precise ways to regulate the whole process [54]. Now turning our attention to yeast, we will highlight several PTMs that have been linked to meiosis, though there are many more in other organisms that can be found in relatively recent review articles and elsewhere [55].

Among others, H3K9ac has been reported in DSB hotspots, and H4K16ac is an important checkpoint for meiotic recombination, which is also facilitated by H4K44ac [56,57,58]. H3K4me3 is linked to recombination initiation and programmed DSB formation, not only in yeast, but also in mouse spermatocytes and oocytes [59,60,61]. H2BK123ub—as well as its homologue in higher eukaryotes, H2BK120ub—participates in meiotic recombination [62]. Moreover, the protection of centromeric cohesion is regulated by H2AS121ph [63].

**Figure 2:**
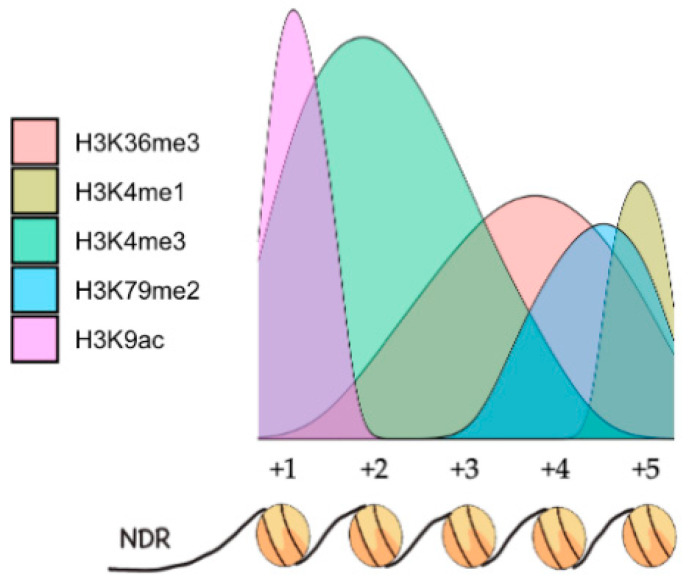
Schematic representation of the distribution of different posttranslational modifications (PTMs) in nucleosomes after the nucleosome-depleted region (NDR), in a hypothetical gene comprised of only five nucleosomes.

Focusing on DSB hotspots, they are characterized by a nucleosome-depleted region (NDR), featuring a noticeable histone mark arrangement directing their activity where H3K9ac is flanking them. After that, H3K4me3 centres on the +1, +2, and +3 nucleosomes. In contrast, H3K4me1/2, H3K36me3, H3K79me2, and H3R2me are found next to the 3’ ends of genes [36,64] (Figure 2).

Due to our research interests, we will discuss in the next subsections (i) the trimethylation of histone H3 on the lysine 4 (H3K4me3), which has been the one most correlated with meiotic DSBs in *S. cerevisiae*, despite being a PTM normally linked to several features of the process of transcription [65,66]; and (ii) the monoubiquitination of histone H2BK123, which has been well studied in transcription regulation and has been implicated in recruiting several DSB factors during meiosis.

### 2.1. H3K4 Trimethylation by the COMPASS Complex Plays an Important Role in Recombination Initiation during Meiosis and DSB Generation

In humans and higher eukaryotes, H3K4me3 is deposited by a family of histone methyltransferases, known as SET1/MLL. It is made up of six different members: SETD1A, SETD1B, and MLL1, 2, 3 and 4 [67]. These complexes present the ability to mark H3K4 with different levels of methylation (that is, H3K4me1, H3K4me2, and H3K4me3), using S-adenosine–methionine (SAM) as donor molecule [68].

In budding yeast, all H3K4 methylation is exclusively mediated by the lysine methyltransferase Set1, by being part of a complex called the Complex of Proteins Associated with Set1 (COMPASS) [68,69] or Set1C [70]. In contrast, the reverse process—namely, de-methylation—is uniquely accomplished by the Jhd2 protein, a subtype of demethylase containing a Jumonji domain. This factor cooperates with COMPASS in order to finely regulate chromatin dynamics and ensure a correct H3K4me3 distribution [71,72].

The COMPASS complex consists in yeast of eight subunits, being the catalytic subunit Set1 (containing an active C-terminal SET domain–Su(var)3–9, Enhancer-of-zeste, and Trithorax), as well as seven additional components: Swd1 (Cps50) and Swd3 (Cps30) (necessary for H3K4 mono-, di-, and trimethylation), Swd2 (Cps35), Sdc1 (Cps25), Bre2 (Cps60) (required for di- and trimethylation), Spp1 (Cps40) (required solely for trimethylation), and a little subunit, Shg1, of which little information is available [67,70].

**Figure 3:**
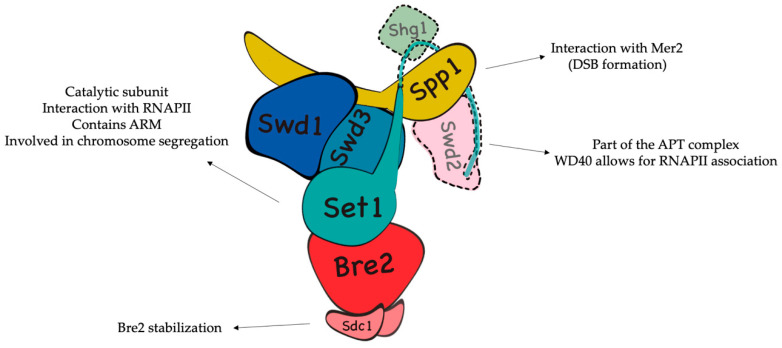
The Complex of Proteins Associated with Set1 (COMPASS). The subunit arrangement model shown here is inspired from the cryo-electron microscopy (EM) structure published by Qu et al. in 2018 [74], which featured a truncated version of Set1, without the N-terminal. This region, as well as Shg1 and Swd2 subunits, have been added to the model (dotted lines). In red (Bre2, Sdc1, and Swd) are the subunits necessary for H3K4me2/3; in blue (Set1, Swd1, and Swd3) are the subunits necessary for H3K4me1/2/3; and in yellow is Spp1, which is necessary for H3K4me3. Little is known about Shg1. The structure is accompanied by several processes in which COMPASS subunits participate.

Two different studies published in 2018 shed light into its structure and mechanisms [73,74]. According to these works, the catalytic module (CM) of COMPASS is organized by Swd1, which has a long C-terminal tail able to surround the other three subunits: Swd3, Bre2, and Set1. Swd3—responsible for the regulation of the trimethylating activity—is situated just beside Swd1, forming something like a set of “arms” (Figure 3).

In the opposite part of the complex, Bre2 and Sdc1 form a trimeric subcomplex, with one copy of the first and two of the latter. This dimerization of Sdc1 is necessary for the correct stabilization of Bre2. Spp1, Swd2, and Shg1, on the other hand, can be found interacting with the N-terminal SET domain and the RNA-recognition motif (RRM) of Set1 [75,76]. Since the cryo-EM structure resolved by Qu et al. [74] contains a truncated version of Set1 (762-1080 aa), neither its N-terminal tail, nor Swd2 or Shg1 appear in it. Strikingly, Swd2 is also associated with the Pta1 (APT) complex, as part of the cleavage polyadenylation factor [77,78,79].

The COMPASS plays an important role in recombination initiation during meiosis and DSB generation. Though originally described as a complex involved in transcription, the identification of Spp1 in these events opened up this new function for COMPASS. Spp1 is a subunit of COMPASS that contains a plant homeodomain (PHD) finger and is able to physically interact with H3K4me3/2, as well as with the Mer2 protein (from the RMM subcomplex) [60]. The interaction between Spp1 and Mer2 enables their anchoring to DSB hotspots, which conducts DSB formation with dependence on Spo11 [60,80]. Spp1 functions are, therefore, not limited to regulating the catalytic activity of COMPASS, since this protein is also able to recognize the methylation state of H3K4 and act in consequence in other processes, such as meiotic recombination. For this reason, Spp1 can be found both around actively transcribed genes and in chromosome axial sites, with independence from Set1 [81]. Furthermore, *set1∆* strains display a general reduction in most DSBs, which has been seen to correlate with H3K4me3 levels [82,83]. This particular strain has also been reported to present a synthetic defect with Rec114 of the RRM subcomplex [84]. Moreover, when Spo11 is targeted to either hot or cold recombination sites, it is unable to induce DSBs on its own [85]. These results suggest that the role played by H3K4 methylation in DSB formation is not limited to simply recruiting the endonuclease.

A conserved proof that there is a relationship between H3K4me3 and meiotic-driven DSBs can be found in mammals, where the histone methylase Prdm9 leads DSBs to occur in DNA motifs recognized by its zinc finger domain [86].

### 2.2. H2B Ubiquitination Is Important for DSB Formation

Monoubiquitination of histone H2B on its lysine 123 (K120 in higher eukaryotes; from this point on, H2Bub) is controlled by a coordinated action of the E2-conjugating enzyme Rad6, the E3 ligase Bre1, and the regulatory cofactor Lge1 [87,88]. Meanwhile, in yeast H2B deubiquitination is performed by two proteases: Ubp10 and Ubp8. Interestingly, Ubp8 belongs to the SAGA complex, and in particular to its deubiquitination module (DUBm) [89,90]. H2B ubiquitination also requires the participation of other factors and complexes like, for instance, PAF1c [91,92] and FACT (facilitates chromatin transcription) [93], which will be addressed later.

In addition to its role in gene expression (see later), H2B ubiquitination has also been related to the formation of DSBs [94]. The ubiquitination of H2B leads to chromatin relaxation, which, in the context of meiosis, enables the recruitment of several DSB repair factors to their proper positions at hotspots [94,95,96]. As a matter of fact, the involvement of H2Bub in chromatin relaxation is evolutionarily conserved from yeast to mammals [95]. Notably, the Rad6 homolog in mammals, *HR6B*, has been related to defects in male fertility when it is depleted in mice, due to errors during spermatogenesis. The function of Rad6 is thus thought to be involved with both the synaptonemal complex and recombination in meiosis, highly implying a role in chromatin remodelling [97]. Likewise, Bre1 and Lge1 proteins have also been related to meiotic processes; *lge1∆* and *bre1∆* cells initiate meiotic DNA replication in the S-phase, later than the wild-type, and take much longer to complete it, presenting a reduced DSB formation as well [62,98].

Interestingly, not only does the PAF1c play a crucial role during transcription, but also its component Rtf1 has been described as participating in the meiotic process [99]. Rtf1 is important for the formation of DSBs, and this role is independent of the presence of Set1. In these lines, modifications of Dam1, the main subunit of kinetochore DASH complex, depend on PAF1c [100]. Dam1 is methylated by Set1 for proper chromosome segregation, and deletions in Paf1, Bre1, Rad6, or Ubp8 (factors implicated in H2Bub) hinder Dam1 methylation.

Furthermore, Ubp8 from SAGA DUBm is involved in H2B and Cse4 deubiquitination [101]. Cse4 is a centromeric, H3-like histone protein (vertebrates’ centromere protein A or CENP-A orthologue). The ubiquitination of Cse4 regulates its localization to centromeres, where the spindle pole body is attached to the chromosome [102]. However, the functional connections between the SAGA DUBm and meiosis are yet to be described. In addition, cells lacking Sgf73, another component of the SAGA DUBm [103], show problems in DNA replication prior to meiosis, and present a reduced expression of *IME1* [98].

## 3. H3K4me and H2Bub Are Crucial Histone Marks during Transcription: Structural Insights into the Molecular Mechanism behind H3K4me/H2Bub Coordination

### 3.1. H3K4me during Transcription

As noted earlier, H3K4 methylation has been well studied in the context of transcription. H3K4me3 is strongly correlated to transcription activation and active genes, with a prominent peak around the transcription start sites (TSSs) being a feature of its distribution. In addition, this peak has been linked in magnitude to the amount of mRNA for a given gene [104,105]. It is in these TSSs where H3K4me3 serves as a binding site for complexes that initiate transcription in promoters, such as the transcription factor II D (TFIID) or the SAGA complex [106]. However, H3K4me is not only related to transcription activation. This mark is also associated with repression by means of antisense transcription [107]. H3K4me2 and me3 peak around the 3’ ends of COMPASS-repressed genes (such as *PHO84*), leading to the expression of antisense transcription at the 3’ ends of coding regions. In this study, the authors found that this antisense transcription is promoted by H3K4me3, but is not fully dependent on the mark.

The actual functions for H3K4me in the regulation of transcription, as well as chromatin organization, are not thoroughly known, though it is known that Set1 is mobilized to TSSs by interacting with the RNA polymerase II (RNAPII) when it is phosphorylated at serine 5 of its Rpb1 C-terminal domain (CTD-Ser5P), or via elongation factors [108,109,110]. This modified version of RNAPII is predominant in the first stages of transcription elongation, and its binding to COMPASS allows for high levels of trimethylation at promoter-proximal regions [105]. The association with RNAPII is mediated by the N-terminal domain of Set1, which directly interacts with the Rpb1 CTD, which is connection-dependent on the WD40 domain of the Swd2 subunit [111].

The little impact of this modification on gene expression at the genomic level highlights that the role of methylation on transcription is more complicated than anticipated [71,112]. Although initial investigations have suggested that H3K4me associates with actively transcribed genes [113], RNA-seq experimentation found no change in mRNA levels in steady-state or dynamic conditions when H3K4me3 was eliminated and could serve as a repressive mark [107]. Furthermore, it might seem that H3K4me could be a consequence of transcription, rather than a regulator of it. Either way, extensive work must be carried out in order to ascertain this extent.

The role of COMPASS in transcription can be further understood in light of several recent studies, which show that this complex is able to bind to mRNAs both in vitro and in vivo [114,115]. Not only was the RRM of Set1 found to be important for this process, but also a myriad of surfaces of the other subunits. This RNA-binding capacity was described as fundamental for a correct topology of COMPASS along transcription.

### 3.2. H2B Monoubiquitination during Transcription

H2Bub has been described to have several roles in gene transcription regulation, since it has been seen to correlate to more RNAPII processivity, being enriched in promoter regions. It has been proposed that, upon H2B monoubiquitination, inter-nucleosomal interactions are disrupted [96]. This is in part due to the large size of the ubiquitin moiety (76 aa), as well as to its position on the nucleosomal face. As a result, chromatin compaction relaxation facilitates the recruitment of other components to DNA [116]. In fact, H2Bub improves intra-nucleosomal interactions, aided by other components, such as the histone chaperone FACT [117]. Other studies have also made clear the implications of H2Bub in RNAPII stalling, in the event where there are DNA lesions, by participating in a transcription-coupled repair pathway [118]. It has been demonstrated that H2Bub stimulates FACT activity to allow the displacement of the H2A/H2B dimer, which permits RNAPII to continue transcription [93].

H2B ubiquitination also requires the presence of another complex in yeast: PAF1c. This complex is comprised of five different subunits: Rtf1 (the most important subunit, containing the termed histone modification domain, HMD), Cdc73, Paf1, Ctr9, and Leo1 [119]. PAF1c is recruited to chromatin by FACT (as shown in mammalians), which is able to recruit enzymes that ubiquitinate H2B (Rad6 and Bre1) [93]. PAF1c is important for the regulation of RNAPII-transcription elongation by interacting with COMPASS and factors involved in termination [120]. PAF1c has also been related to the phosphorylation state of the C-terminal domain of Rpb1, a promotion of H3K36 trimethylation, as well as histone acetylation on active genes [121].

### 3.3. Structural Overview of COMPASS Activation upon H2B Ubiquitination

**Figure 4:**
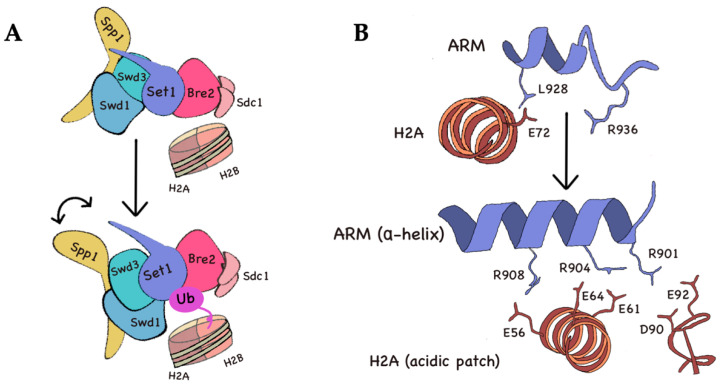
Structural overview of COMPASS upon H2B ubiquitination (**A**) Change of conformation of the COMPASS complex upon ubiquitylation of H2B. Swd1, Swd3, and Spp1 subunits rotate away from the rest of the catalytic module of COMPASS; Swd1 establishes a series of contacts with the ubiquitin that strengthen the interaction and stabilize the conformational change; (**B**) Schematic representation of the arginine-rich motif (ARM) stabilization. The ARM is stabilized by the contact with the ubiquitin, and is able to interact with several residues of the acidic patch of H2A, after having adopted an α-helix conformation, according to Worden et al. [127]. Upon the conformational change adopted by the ARM, the residues participating in the interaction with the acidic patch vary, as indicated.

Notably, H3K4me needs H2Bub in order to occur, since *rad6∆* cells are devoid of methylation [122] and both modifications are highly associated to transcription [104,123]. This kind of interdependency is commonly referred to as “cross-talk”, where a histone modification serves as template for the pattern of a second one. Unfortunately, the mechanisms whereby this crosstalk is mediated have been largely unknown, yet several recent structural studies seem to help us understand this mystery. The yeast version of COMPASS presents a catalytic module (CM) able to embrace an H3 tail and made up of five subunits: Set1 (SET domain), Swd1, Swd3, Sdc1, and Bre2 [124]. This module seems to display a component impairing the methyltransferase activity of the complex whenever a H2B-modified nucleosome is absent [73]. However, the presence of the ubiquitin moiety does not modify the affinity of COMPASS for the nucleosome, but is likely to activate the enzyme by inhibiting the CM impairment [125]. Once ubiquitin has been conjugated to H2B, it locates to a cleft between Swd1, Set1, and Bre2 (Figure 4A). The moiety presents several surfaces interacting with COMPASS, including a definite interface comprised of a hydrophobic patch (I44-V70-L8-H68, namely the I44 patch) that contacts a hydrophobic patch consisting of both the N-terminal and C-terminal tails of Swd1, which change their conformation [125,126,127]. This contact is later stabilized by a salt bridge between Lys48 and Arg42 of the ubiquitin and Glu15 and Glu405 of Swd1. Such is the importance of this subunit in ubiquitin recognition that a *swd1* deletion completely abolishes any kind of H3K4 methylation by COMPASS [127].

The main inhibitory region of the COMPASS CM is concentrated on an arginine-rich motif (ARM) that immediately precedes the SET domain of Set1 [128] (Figure 4B). The ARM is found disordered, without a ubiquitinated nucleosome [74]; upon binding to H2Bub, it folds and is constrained into the nucleosomal acidic patch provided by H2A, and adopts an α-helix conformation [125,128]. The anchoring of this ARM into the acidic patch enables the connection between the catalytic SET domain and the ubiquitinated H2B, as well as its closeness to Swd3, Swd1, and Bre2 subunits. This binding also permits a sound restructuration of a Set1 α-helix (residues 926 to 933) into an extended strand, parallel to the ARM; alleviating a steric hindrance in Set1 [127] (Figure 4B). Notably, even though many conformational changes are described in Set1, none of them directly affect the SET catalytic domain. Consequently, it is highly likely that the increased activity is due to the contact to the nucleosome provided by the ARM [125,127].

In summary, these new results help us better understand the molecular mechanism behind the coordination between H2Bub and H3K4me. Further investigations to assess whether this mechanism occurs both in transcription and meiotic recombination and at different genomic positions are required to fully understand the H2Bub/H3K4me connection.

## 4. Human Diseases Associated to Defects in H3K4me and H2Bub Machineries

Even if it has been stated several times through this work, a well-defined trait characterizing all aforementioned mechanisms is the fact that they are extensively conserved across evolution. Actually, most of the mentioned factors present well-described orthologues in higher eukaryotes that have been involved in numerous disorders, mainly due to the crucial nature of the processes they regulate. A very short summary of some of the diseases associated to mutations in these factors are given below, and are recapitulated in Table 1 (more extensive reviews can be found in the literature [129,130,131,132,133,134]).

Set1/MLL has been reported to participate in cancer and ageing [67], being a hallmark for both acute lymphoblastic and acute myeloid leukemia [130], as well as in hematopoiesis [136]. Defects on MLL2/Set1 (also referred to as KMT2D in mammals) trigger the rare autosomal dominant Kabuki syndrome 1 [135]. MLL3 and MLL4 have been connected to cancer development, owing to interactions with p53 [143], which has led to use of them as potential targets in treatments against leukemia [144,145,146]. Other approaches are related to MLL1/MLL2 translocation problems [147].

As stated before, Set1/MLL is implicated in lifespan and ageing as well. High levels of H3K4 methylation are undesirable for an extended longevity, where reactive oxygen species (ROS) play a vital role [67,148]. An increase in ROS is related with a H3K4me3 reduction regulated by Set1, as observed in *C. elegans* and in yeast [149].

Furthermore, Set1 is required for proliferation of ESCs, iPSCs, and neuronal stem cells. Besides participating in leukemia, MLL1/MLL2 are indispensable in embryogenesis [130]. A Set1/COMPASS subunit required for H3K4me3 possesses the CXXC zinc finger protein 1 (CXXC1 or Cfp1, a Spp1 yeast ortholog), which binds to unmethylated CpG islands [150]. CXXC1 has been described as having a pivotal function in oocyte development [151].

As described here, recombination hotspots are triggered by PRDM9-mediated H3K4me3. Deficiencies in this gene are associated with defective synapses and male infertility [140,141]. CXXC1 also interacts with PRDM9 in spermatocytes, but it is not essential to form DSBs [152].

Among others, in the case of H2B ubiquitination, Rad6 (HR6B in mammals) defects result in male infertility [97,134], and Rad6 is overexpressed in ovarian cancer [142]. Apart from PAF1c’s involvement in meiosis and H2Bub, it is also correlated with cancer development [153,154,155]. The Paf1 subunit interacts with CXXC1 and PD2 (pancreatic differentiation 2), and is upregulated in ovarian and pancreatic stem cells [156]. PAF1c is also implicated in parathyroid carcinoma (PC) [137], ESC pluripotency maintenance [138], and mitochondrial autophagy [139].

## 5. Future Directions and Concluding Remarks

All chromatin-dependent functions are regulated by epigenetic modifications. Among them, histone modifications constitute one of the best-studied regulatory elements that orchestrate specific patterns that will modulate from gene expression to meiosis. The in-depth study of these modifications has provided an extremely interesting view of how different histone modification patterns could lead to the renovation of chromatin structures that allow the signalling required to trigger a specific process. Notably, most of these modifications are executed by multi-subunit complexes that can accomplish different functions depending on their partners and the environment. Our knowledge of how specific histone modifications—for instance, the focus of this review: H3K4me and H2Bub—occur upon a precise signal is far from being complete.

Many questions remain unanswered—for instance, (i) how can the histone writers and erasers differentiate between genomic loci? (ii) How can the histone readers discriminate between the distinct chromatin-based processes? (iii) Which is the interconnection between specific histone modifications that regulates different biological processes? (iv) Are the molecular mechanisms behind each modification shared between molecular events? (v) Is there a set of specific factors that regulate the writers and the erasers only during meiotic recombination? An interesting possibility is that the capacity of different subunits to bind to different partners enables them for specific roles. As a matter of fact, the presence of Spp1 in both processes might indicate its implications in both of them and how they are linked, given that it is localized to COMPASS, allows the deposition of the H3K4me3 mark, and when forming a subcomplex with Mer2, is able to read the mark and conduct DSB formation [59,81,82]. Its presence as differentiated pools might highlight its role as an important interplayer in meiosis and transcription.

Much effort has been focussed on addressing the role of the histone H3K4me and H2Bub writers (COMPASS and Rad6/Bre1/Lge1, respectively); meanwhile less is known about their erasers (Jhd2 and Ubp8/Ubp10, respectively). Jhd2 has been better studied, indicating that it is a general regulator, being essential for delaying transcriptional quiescence during sporulation. In fact, *jhd2∆* cells show a precocious gametogenesis and stress-sensitive spores [157,158]. Additionally, Jhd2 is known to act with COMPASS to ensure a symmetrical H3K4 methylation, and its demethylase activity seems to be hindered by Spp1 [72,81]. In contrast, less is known about the role of Ubp8 or Ubp10 in H2B deubiquitination during meiosis. Interestingly, the enzymatic activity of Ubp8 is carried out as part of the SAGA complex [159,160,161]. SAGA also has the ability to acetylate histones through the activity of Gcn5 [21], which is of extraordinary relevance for transcription activation and the expression of early genes indispensable for entrance into the premeiotic S phase (see above) [98]. Notably, in *S. pombe* the control of a master regulator of cell fate decision, Ste11, depends on different activities of the SAGA complex for the switch from mitotic growth to sexual differentiation [162]. Is Ubp8 activity also essential for early gene activation? Does SAGA participate in meiotic recombination through the coordinated activity of Gcn5 and Ubp8? Future investigations will shed light into these possible roles for SAGA during meiosis.

**Figure 5:**
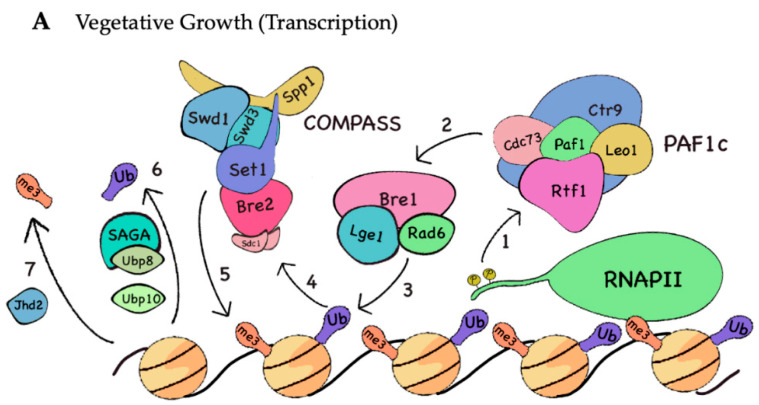

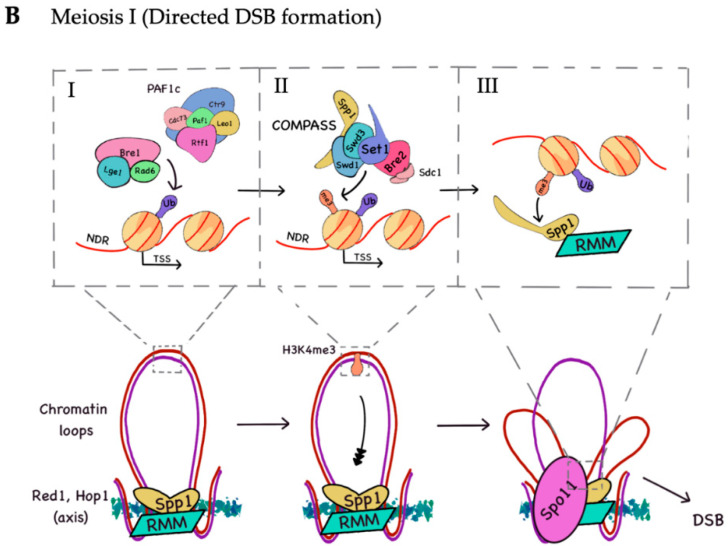
Role of H2Bub and H3K4me3 in transcription and DSB formation. (**A**) General overview of the cross-talk. PAF1c recognizes RNA polymerase II (RNAPII) phosphorylated on the S2 and S5 of its CTD (1), and promotes H2B ubiquitination by Bre1–Lge1–Rad6 (2,3). This modification is recognized by the COMPASS complex (4), which, in turn, trimethylates H3 on its lysine 4 (5). Conversely, H2Bub is removed by the ubiquitin-proteases Ubp10 and Ubp8, the latter belonging to the DUBm of SAGA (6). Trimethylation is eliminated by Jhd2 demethylase (7); (**B**) Spp1 is situated on the chromosome axis, interacting with the RMM subcomplex. In the chromatin loop, the first nucleosome after a nucleosome-depleted region (NDR), situated around the transcription start site (TSS) of a gene, is ubiquitinated by Bre1/Rad6, in coordination with PAF1c (I). H2Bub is read by COMPASS (also containing Spp1), which is now able to methylate H3K4. This H3K4me3 mark on the nucleosome begins to be attracted and protected by COMPASS-free Spp1 situated on the chromosome axis (II). The NDR becomes closer to the chromatin axis, thanks to the interaction with Spp1, and Spo11 is recruited, giving rise to DSBs (III).

Everything that has been described here brings our attention to the profound relationship between H3K4me and H2Bub, and the need for understanding more deeply how the machineries that write, read, and erase them are able to discern between the different molecular events occurring in chromatin (Figure 5).

## Figures and Tables

**Table 1:** List of some human diseases associated to machineries involved in H3K4me3 and H2Bub.

Complex.	Pathologies Associated	Reference
COMPASS/Set1	Lymphoblastic and acute myeloid leukemia	[130]
	Kabuki syndrome 1	[135]
	Hematopoiesis	[136]
	Embryonic stem cell (ESC), induced pluripotent stem cell (iPSC) and neuronal stem cell proliferation	[130]
	Embryogenesis	[130]
PAF1c	Parathyroid carcinoma (PC)	[137]
	ESC pluripotency maintenance	[138]
	Mitochondrial autophagy	[139]
PRDM9	Defective synapsis and male infertility	[140,141]
Rad6	Male infertility	[97,134]
	Ovarian cancer	[142]

## Abbreviations

DSB	Double-strand break
PTM	Posttranslational modification
COMPASS	Complex of Proteins Associated with Set1
PAF1c	RNA polymerase II associated factor 1 complex
cryo-EM	Cryo-electron microscopy
RRE	Rme1 repressor element
lncRNA	Long non-coding RNA
APC/C	Anaphase-promoting complex/cyclosome
SAGA	Spt-Ada-Gcn5 acetyltransferase
CO	Crossover
NCO	Non-crossover
RMM	Mer2-Mei4-Rec114
MRX	Mre11-Rad50-Xrs2
SC	Synaptonemal complex
CE	Central element
LE	Lateral elements
SIC	Synapsis initiation complex
SPB	Spindle pole body
NDR	Nucleosome-depleted region
SAM	S-adenosine-methionine
CM	Catalytic module
RRM	RNA-recognition motif
APT	Associated with Pta1
PHD	Plant homeodomain
DUBm	Deubiquitinating module
FACT	Facilitates chromatin transcription
CENP-A	Centromere protein A
TSS	Transcription start site
TFIID	Transcription factor II D
RNAPII	RNA polymerase II
CTD	C-terminal domain
HMD	Histone modification domain
ARM	Arginine-rich motif
ESC	Embryonic stem cell
iPSC	Induced pluripotent stem cell
PC	Parathyroid carcinoma
ROS	Reactive oxygen species
PD2	Pancreatic differentiation 2
